# Comparison of three different methods for the quantification of equine insulin

**DOI:** 10.1186/s12917-016-0828-z

**Published:** 2016-09-09

**Authors:** T. Warnken, K. Huber, K. Feige

**Affiliations:** 1 Department of Physiology, University of Veterinary Medicine Hannover, Foundation, Bischofsholer Damm 15, 30173 Hannover, Germany; 2 Institute of Animal Science, Faculty of Agricultural Sciences, University of Hohenheim, Fruwirthstraße 35, 70593 Stuttgart, Germany; 3 Clinic for Horses, University of Veterinary Medicine Hannover, Foundation, Bünteweg 9, 30559 Hannover, Germany

**Keywords:** Horse, Equine, Insulin, Quantification, ELISA, RIA, CLIA, EMS

## Abstract

**Background:**

Exact analysis of equine insulin in blood samples is the key element for assessing insulin resistance or insulin dysregulation in horses. However, previous studies indicated marked differences in insulin concentrations obtained from sample analyses with different immunoassays. Most assays used in veterinary medicine are originally designed for use in human diagnostics and are based on antibodies directed against human insulin, although amino acid sequences between equine and human insulin differ. Species-specific assays are being used more frequently and seem to provide advantages compared to human-specific assays. The aim of this study was to compare three immunoassays, one porcine-specific insulin enzyme-linked immunosorbent assay (ELISA), advertised to be specific for equine insulin, one porcine-specific insulin radioimmunoassay (RIA) and one human-specific insulin chemiluminescence immunoassay (CLIA), all three widely used in veterinary laboratories for the analysis of equine insulin. Furthermore, we tested their clinical applicability in assessing insulin resistance and dysregulation by analysis of basal blood and blood samples obtained during a dynamic diagnostic stimulation test (OGT) with elevated insulin concentrations.

**Results:**

Insulin values obtained from the ELISA, RIA and CLIA, investigated for analyses of basal blood samples differed significantly between all three assays. Analyses of samples obtained during dynamic diagnostic stimulation testing with consecutively higher insulin concentrations revealed significantly (*p* < 0.001) lower insulin concentrations supplied by the CLIA compared to the ELISA. However, values measured by ELISA were intermediate and not different to those measured by RIA. Calculated recovery upon dilution, as a marker for assay accuracy in diluted samples, was 98 ± 4 % for ELISA, 160 ± 41 % for RIA and 101 ± 11 % for CLIA.

**Conclusions:**

Our results indicate that insulin concentrations of one sample measured by different methods vary greatly and should be interpreted carefully. Consideration of the immunoassay method and reliable assay-specific reference ranges are of particular importance especially in clinical cases where small changes in insulin levels can cause false classification in terms of insulin sensitivity of horses and ponies.

## Background

The equine metabolic syndrome (EMS) has attracted increasing attention in equine veterinary practice in recent years and is becoming more common due to today’s horse husbandry and feeding practices. Different diagnostic tests to determine dysregulation of glucose and insulin homeostasis in horses are currently used to diagnose EMS. Tests are based on quantitative measures either of basal serum insulin concentration in fasted and non-fasted horses or of increased insulin concentration stimulated by oral [1, 2] or intravenous [3] dynamic diagnostic tests. Complex hyperinsulinaemic euglycaemic clamp (HEC) tests, which were considered to be the gold standard for assessment of insulin resistance (IR) [4], were usually reserved to research approaches due to their complex and expensive implementation. High insulin indicative of IR or insulin dysregulation (ID) in EMS horses is one of the features associated with this metabolic disorder [5]. Exact pathophysiology resulting in IR is not known to date. Several hypothesis leading to the clinical signs have been discussed in literature. Increased insulin degradation or neutralization, decreased binding of insulin to its receptor, as well as impaired downstream signaling were described. Whereas the term IR used in context with EMS is mainly characterized by reduced tissue response of insulin-dependent tissues the newly introduced term ID is used to describe in summary abnormalities of insulin metabolism [5]. Therefore, an exact quantification of insulin by laboratory analysis is the most important and challenging step to diagnose EMS-related changes in insulin regulation. Several immunoassay methods for the quantification of insulin in equine serum or plasma samples are commercially available. However, due to the use of different methods for quantifying equine insulin, discrepancies between results obtained from different studies have occurred [6, 7]. Since no assay with a specific antibody against equine insulin is available, most of the commercial immunoassays are based on antibodies directed against human or non-equine insulin. Only one enzyme-linked immunosorbent assay (ELISA) advertised by the manufacturer as being specific for measuring equine insulin is commercially available^1^, and this is based on anti-porcine insulin antibodies. This assay has been validated for use in horses [8] and has already been used successfully in several studies [9, 10].

The aim of our study was to compare the results of this equine-optimized porcine-specific ELISA with results obtained from a radioimmunoassay^2^ (RIA) and a chemiluminescence immunoassay^3^ (CLIA) for measurements of equine insulin in serum samples. Furthermore, the second aim of the study was to evaluate the applicability of the three assays for samples obtained from horses and ponies under fasted conditions and from horses and ponies with stimulated insulin secretion during diagnostic procedure.

## Methods

### Animals and samples

Forty blood serum samples were collected from seventeen horses and ponies of different breeds, age (14 ± 6 years), weight (478 ± 179 kg) and body condition score (6.8 ± 1.1) to obtain samples with a broad range of insulin concentrations. Blood samples from healthy, university-owned horses were collected during a study, which has been approved by the ethics committee within the University of Veterinary Medicine, Hannover, and the State Office for Consumer Protection and Food Safety in accordance with the German Animal Welfare Law (LAVES– Reference number: 33.14 42502-04-13/1259). Blood samples from insulin dysregulated horses and ponies were collected during routine diagnostic procedures in the Clinic for Horses and informed consent was obtained from the clients for publication. Blood samples were collected after fasting the horses overnight (basal, *n* = 20), and additionally after stimulation by an oral glucose testing (OGT, *n* = 20) procedure [2]. OGT was carried out by administering 1 g/kg btw glucose powder dissolved in two liters water by naso-gastrical-tubation. Samples were collected via jugular vein catheter, transferred into plain tubes^4^ for serum preparation and were incubated at room temperature for 1 h, centrifuged at 3000 g for 6 min and serum was stored at −80 °C. Prior to analyses samples were thawed once and split into different aliquots containing the required volume for each assay. Afterwards, they were re-frozen and sent on dry ice to the laboratories.

### Assays

The porcine-specific insulin assay^1^ is a solid phase two-side sandwich ELISA optimized for the quantification of equine insulin in serum or plasma. It is based on monoclonal mouse anti-porcine insulin antibodies coupled to plate and free monoclonal mouse anti-porcine insulin antibody conjugate labeled with horseradish peroxidase. Porcine insulin is used for calibrators to compute calibration curve via cubic spline regression. The limit of detection for this assay is 1.17 μIU/mL, but concentrations of samples with absorbance below the lowest calibrator (2.34 μIU/mL) should not be calculated. Cross reactivity of the assay is stated at 100 % for porcine insulin and 22 % with human insulin (according to the manufacturer’s protocol) (Table 1).

**Table 1:** Information about the three assays examined for quantification of equine insulin

Assay	Standards	Primary antibody	Second antibody
Mercodia Equine Insulin ELISA	Porcine insulin	Mouse monoclonal anti-porcine insulin	Mouse monoclonal anti-porcine insulin HRP-conjugate
Millipore Porcine Insulin RIA	Human recombinant insulin	Guinea pig anti-porcine insulin	Goat anti-guinea pig IgG
Siemens ADVIA Centaur Insulin Assay	-^a^	Monoclonal mouse anti-insulin AE-conjugate	Monoclonal mouse anti-insulin coupled to paramagnetic particles

^a^No information available

For sample analysis by RIA, a porcine-specific one-site insulin RIA^2^ was used. This assay is based on ^125^I-labeled insulin and used guinea pig anti-porcine insulin antibodies and goat anti-guinea pig IgG antibodies. Purified human recombinant insulin preparations were used as standards for calibration. The manufacturer’s protocol referred to the assay’s limit of linearity of 200 μIU/mL and recommended diluting the samples which have greater concentrations with the assay buffer. The limit of detection for this assay is 1.61 μIU/mL when 100 μl of sample is used. The cross-reactivity of the assay is stated at 100 % for porcine insulin, 100 % for human insulin and 90 % for bovine insulin. No information about cross-reactivity for equine insulin is given by the manufacturer. Measurements with the RIA were performed by a commercial veterinary endocrinology laboratory.

A human-specific insulin chemiluminescent immunoassay^4^ with two-site sandwich technique and direct chemiluminescent technology was used for the sample analysis by CLIA. The assay is based on monoclonal mouse anti-insulin antibodies and chemiluminescent detection with monoclonal mouse anti-insulin antibodies labeled with acridinium ester. The limit of detection of this assay is 0.5 μIU/mL and the measuring range is from 1.0 to 300 μIU/mL. No information about cross-reactivity for equine insulin is given by the manufacturer. Measurements using the CLIA were performed by a commercial veterinary laboratory. Due to the fact that all tested assays use no equine standards, all the assays only provide an approximation of equine insulin concentrations.

### Revalidation of the equine-optimized porcine-specific insulin ELISA

Intra-assay CV was calculated by division of the standard deviation by the corresponding mean of 15 replicates of equine serum samples with low (mean: 2.34 μIU/mL) and medium (mean: 108.81 μIU/mL) insulin concentrations. Inter-assay CV was calculated by division of the standard deviation by the corresponding mean of replicate samples, each measured on 25 different plates. Commercial controls^5^ with low (5.03 μIU/mL), medium (18.84 μIU/mL) and high (60.84 μIU/mL) insulin concentrations (classifications according to manufacturer), and equine serum samples with low (mean: 10.53 μIU/mL) and medium (mean: 109.98 μIU/mL) insulin concentrations were used for the calculation of concentration-dependent inter-assay CVs. The recovery upon dilution (RUD) and the linearity of dilution were calculated to prove accuracy. A sample selected with a medium insulin concentration (99.45 μIU/mL) was measurable without any dilution and in five different dilution steps up to the 1:40 ratio dilution within the calibration range of the assay. Sample buffer^6^ was used for dilution and at least 40 μl of the equine serum sample.

### Comparison of methods

Basal serum samples and simulated samples from the OGT procedure were measured undiluted in all three assays for comparison of methods. Moreover, stimulated samples were additionally measured diluted in a ratio of 1:4 with sample buffer^6^ to calculate the RUD for each assay to prove accuracy in the measurement of dilution procedure. The RUD was calculated as a percentage recovery of the insulin concentration in the diluted sample related to the corresponding undiluted samples. The RUD analyses were performed without the knowledge of the laboratories commissioned.

### Stability of insulin after freezing, thawing and eight weeks of storage

In order to investigate the effect of repeated freezing and thawing on the stability of insulin in serum samples measured by equine-optimized, porcine-specific ELISA, 33 samples were thawed, measured and refrozen at −80 °C. Eight weeks later, samples were thawed at ambient temperature for 1 h and measured again. Samples covering a broad range of insulin concentrations were chosen to match the assay’s analytical range from 2.34 to 175.5 μIU/mL. The samples were subdivided into three subgroups of low (3.51–15.21 μIU/mL), medium (30.42–90.09 μIU/mL) and high (92.43–125.19 μIU/mL) concentrations with eleven samples in each subgroup.

### Statistics and calculations

Data analysis was performed using GraphPad Prism software^7^. The Shapiro-Wilk normality test was used to assess the normality of data distribution. Wilcoxon matched-pairs signed rank test, Spearman correlation and Deming regression analyses were used to compare results from different assays and to verify relationships between the three methods. In cases of assay-based non-detectable insulin concentrations, the corresponding samples were excluded from the statistics. Bland-Altman analysis was performed to calculate method-dependent bias and limits of agreements. Wilcoxon matched-pairs signed rank test was also used to examine the effect of repeated freezing and thawing on the stability of equine insulin. Statistical significance was set at *p* < 0.05.

## Results

**Fig. 1:**
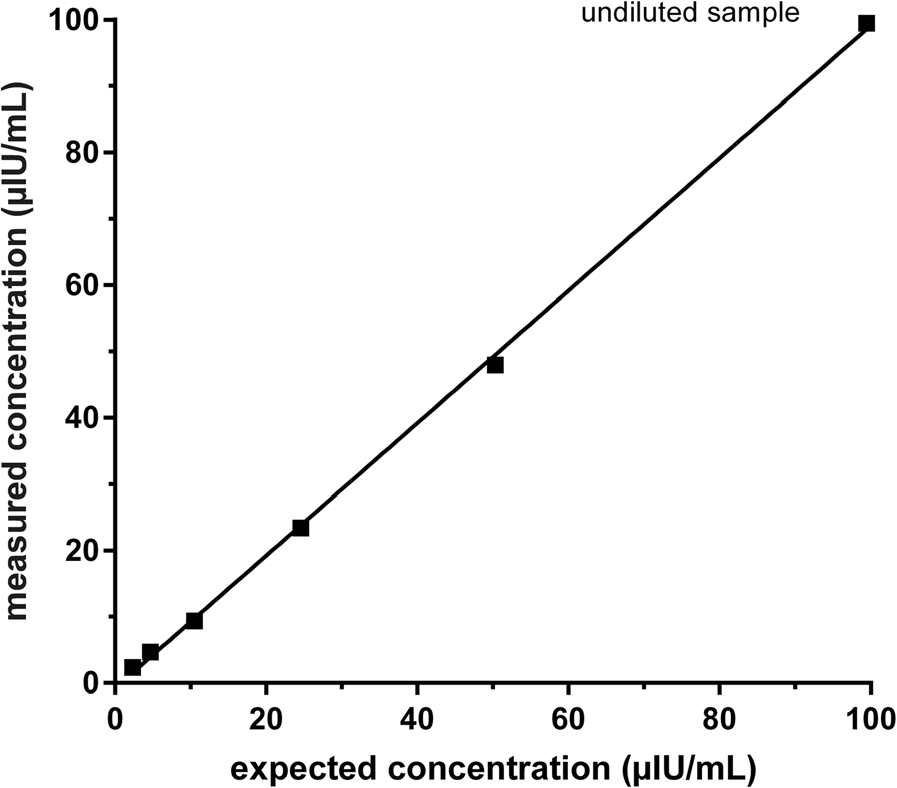
Linearity upon dilution in porcine-specific insulin ELISA. Linear regression analysis indicates best-fit line of *Y* = 0.9987 x – 0.7385 (*r* ^*2*^ = 0.9994, *P* = < 0.0001)

In re-validation experiments, intra-assay CV was 4.61 % at low insulin concentrations (2.34 μIU/mL) and 1.91 % at medium insulin concentrations (108.81 μIU/mL) using the equine-specific insulin ELISA. The inter-assay CV was 5.27 %, 3.24 % and 3.17 % for the low (reference value 5.03 μIU/mL insulin), medium (reference value 18.84 μIU/mL insulin) and high (reference value 60.84 μIU/mL insulin) commercial controls, respectively. Inter-assay CVs for equine serum samples were 7.34 % and 4.83 % for low (mean: 10.53 μIU/mL) and high concentrations (mean: 109.98 μIU/mL), respectively. The RUD in the first revalidation experiment was 95 % (range from 94 to 96 %), calculated in five dilution steps. The linearity upon dilution was excellent (*r*^*2*^ = 0.9994; *p* < 0.0001), showing a strong relationship between the calculated and measured concentrations of the diluted samples (Fig. 1).

**Fig. 2:**
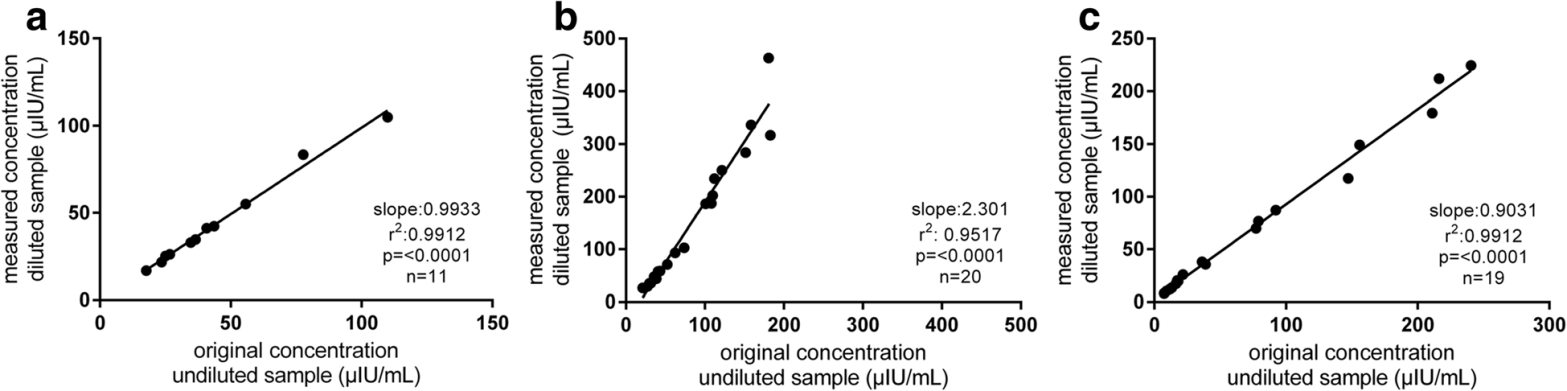
Recovery upon dilution (RUD) of equine insulin in the three assays investigated: **a** ELISA (98 ± 4 %), **b** RIA (160 ± 41 %) and **c** CLIA (101 ± 11 %). The RUD was calculated as percentage recovery of the insulin concentration in the 1:4 diluted sample related to the corresponding undiluted sample. Samples were diluted with commercially available sample buffer^6^

The ELISA provided results within the analytical range for 17 of 20 basal samples and for 11 of 20 stimulated samples (Table 2). The analytical range of the assay was from 2.34 to 175.5 μIU/mL (corresponding range in μg/L: 0.02 to 1.5; conversion factor: 117), as stated by the manufacturer. The mean RUD in samples analyzed by ELISA was 98 ± 4 % (Fig. 2a).

**Table 2:** Information about the number of samples which were measureable within the analytical ranges of the three assays investigated, without previously dilution

Assay	Number of samples	
	Basal, fasted samples	Stimulated OGT samples
ELISA	17/20	11/20
RIA	19/20	20/20
CLIA	20/20	19/20

The RIA supplied results for 19 basal samples and for all 20 stimulated samples (Table 2). The analytical range for the RIA was stated as 1.61 to 200.00 μIU/mL. The RUD in the RIA analyses was 160 ± 41 % (Fig. 2b).

One of the stimulated samples from the OGT procedure was not measureable within the analytical range (1.0 to 300 μIU/mL) in the CLIA (reported as > 300 μIU/mL) (Table 2). The RUD in the CLIA analyses was 101 ± 11 % (Fig. 2c).

**Fig. 3:**
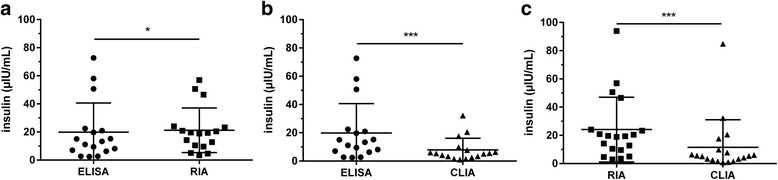
Insulin concentrations measured in basal, fasted blood samples by ELISA, RIA and CLIA. Data were analyzed by Wilcoxon matched-pairs rank test. **a** ELISA and RIA (*n* = 17) **b** ELISA and CLIA (*n* =1 7) **c** RIA and CLIA (*n* = 20). *p < 0.05, **p < 0.01, ***p < 0.001

**Fig. 4:**
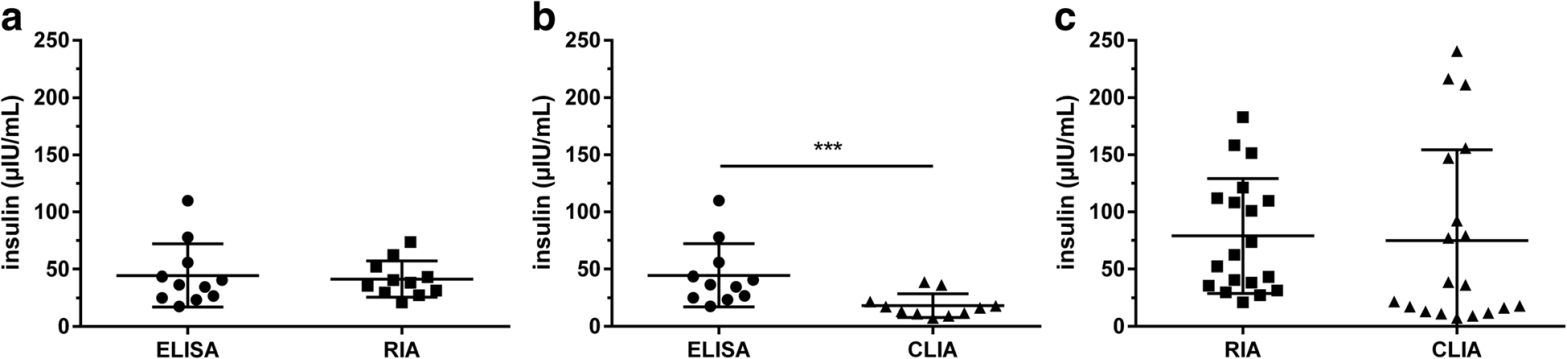
Insulin concentrations measured in stimulated blood samples from OGT procedure by ELISA, RIA and CLIA. Data were analyzed by Wilcoxon matched-pairs rank test. **a** ELISA and RIA (*n* = 11) **b** ELISA and CLIA (*n* = 11) **c** RIA and CLIA (*n* = 19). *p < 0.05, **p < 0.01, ***p < 0.001

**Fig. 5:**
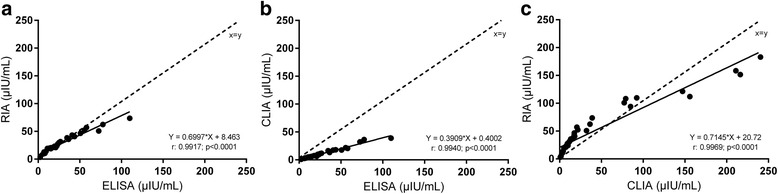
Scatterplot of measurement results for basal and stimulated samples ELISA compared to **a** RIA and **b** CLIA. **c** Scatterplot of results supplied by CLIA compared to RIA

**Fig. 6:**
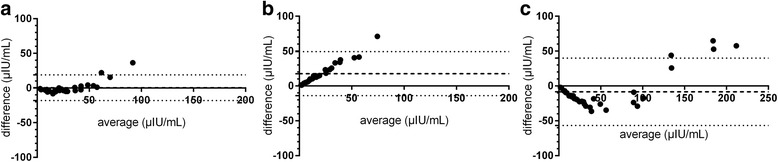
Bland-Altman plot of average compared to difference **a** ELISA minus RIA (*n* = 28), **b** ELISA minus CLIA (*n* = 28) and **c** CLIA minus RIA (*n* = 39). *Dashed line* = bias, *pointed line* = 95 % limits of agreements

A comparison of the results provided by the three methods of analysis of basal, fasted blood samples for equine insulin indicated statistically significant differences (Fig. 3). Results provided by the CLIA for basal blood samples were significantly (*p* < 0.001) lower compared to results supplied by ELISA or RIA (Fig. 3b, c). Furthermore, differences between ELISA and CLIA in stimulated samples were statistically significant (Fig. 4b), whereas results supplied by ELISA and RIA and RIA and CLIA did not differ significantly (Fig. 4a, c). Despite these differences between insulin concentrations measured by ELISA, RIA and CLIA, Spearman correlations and Deming regression analyses revealed significant correlations and linear relations between all three assays tested (Fig. 5a–c). Bland-Altman Plot analyses showed good agreement with a small bias of −0.44 μIU/mL (95 % limits of agreement: −18.12 to 18.99) comparing the results of ELISA and RIA (Fig. 6a), whereas variations in the results supplied by ELISA compared to CLIA (Fig. 6b; bias: 17.65 μIU/mL; 95 % limits of agreement: −13.92 to 49.22) and CLIA to RIA (Fig. 6c; bias: −8.39 μIU/mL; 95 % limits of agreement: −56.76 to 39.98) occurred. Additionally, the range of variation between results from ELISA and CLIA and between CLIA and RIA increased with elevated insulin concentrations (Fig. 6b, c).

The average recovery rate of equine insulin in 33 samples after one cycle of refreezing and thawing was 97 ± 17 %, but the corresponding range of recovery stretched from 71 to 158 %. In total, insulin concentrations were significantly (*p* = 0.0232) lower in the second measurement (54.99 ± 40.95 μIU/mL) compared to the first (58.5 ± 43.29 μIU/mL). When samples were subdivided into concentration range groups of low (3.51–15.21 μIU/mL), medium (30.42–90.09 μIU/mL) and high (92.43–125.19 μIU/mL) concentrations, the range as well as the mean recovery between the three concentration ranges differed, but there was no statistically significant difference between the first and second measurement when samples were clustered in subgroups depending on concentration. The mean recovery was 98 ± 16 % (range: 79 to 129 %) in the low, 99 ± 24 % (range: 74 to 158 %) in the medium and 92 ± 12 % (range: 71 to 107 %) in the high concentration group.

## Discussion

Most immunoassays used in veterinary medicine for analyzing insulin concentrations in equine serum or plasma samples were originally designed for human diagnostics and research, and specific immunoassays for the quantification of equine insulin are not commercially available. A human-specific insulin radioimmunoassay^8^ was used in a variety of equine studies [3, 11, 12] in which several IR and ID diagnostic tests and related reference ranges for plasma and serum insulin had been established. Unfortunately, this assay is no longer available, thus, there is an urgent need to reevaluate the reference ranges established on analyses from samples measured with other approaches for measuring insulin.

### Reproducibility, recovery upon dilution and measuring range of assays

The CVs calculated for the ELISA showed that insulin concentration-dependent differences in CVs occurred and that the CVs calculated by the use of equine serum samples were higher than the CVs stated in the manufacturer’s protocol. Very high or low insulin concentrations could not be detected by all assays due to the various analytical ranges of the assays. Therefore, as samples vary greatly in insulin concentration when obtained in fasted conditions or in dynamic diagnostic tests with induced insulin secretion, selection of an assay with an appropriate analytical range is important for the detection of very high or low insulin concentrations. Moreover, our results show that samples with high insulin concentrations, similar to those that can occur in patients with IR or ID, needed to be diluted in some cases to obtain results. Although the ELISA offered the smallest analytical range of the three assays tested, its excellent RUD results enabled the coverage of a broad range of insulin concentrations. Using this ELISA allowed valid measurements of earlier diluted samples of dynamic diagnostic tests. By contrast, using the RIA, all samples could be measured without dilution in the analytical range of the assay. However, RUD experiments did not provide satisfactory results. In a substantial amount of samples, too high insulin concentrations were obtained for the diluted samples compared to the corresponding undiluted samples. The CLIA offered the widest analytical range when compared to the other two assays, but also could not measure all high insulin concentration samples. This assay, similar to the ELISA, exhibited good RUD results, leading to the conclusion that appropriate dilution of samples in this assay do not falsify results.

### Dilution buffers

We used commercially available sample buffer^6^ to dilute the samples in this study. This buffer was designed for use in the ELISA investigated. Öberg and colleagues [8] evaluated the RUD and linearity of dilution in this ELISA with physiologic saline as a dilution medium in four dilution steps and found similar results regarding linearity, but poorer RUD of 92 to 122 %. Dilution of serum samples with zero calibrator provided in the ELISA yielded higher recovery rates of 102.4 ± 20.5 % in another study [13]. Therefore, dilution of samples with the manufacturer’s commercially available sample buffer^6^ seems to improve results obtained from diluted samples in this assay. Tinworth and colleagues [6] recommended that samples with high insulin concentrations which required dilution should be diluted with charcoal stripped equine plasma instead of the manufacturer’s diluent in order to improve results obtained by analysis with a human-specific insulin RIA^8^ mentioned previously. One limitation in our study is that all our samples have been diluted with commercially available sample buffer^6^ designed for use in the equine-optimized, porcine-specific ELISA and were then used in the different assays. Therefore, results of this RUD analysis may also be impaired by interactions of the dilution medium and assay components. The RUD results may be improved by using an appropriate method-dependent dilution medium.

### Insulin molecule stability

The stability of insulin in blood samples is a commonly discussed issue in both human and veterinary medicine. Optimal pre-analytical handling of samples is essential for valid analytical results, but is often not feasible in the daily routine of an ambulatory practice. The recovery range observed indicates quite variable results. Some of the refrozen and thawed samples measured had a lower insulin concentration in the second analysis, whereas others had higher insulin concentrations. Parts of the variations and effects between the first and second measurement may be due to inter-assay variations, but, as already presented, inter-assay CVs for low equine insulin concentration samples and high insulin concentration samples in the ELISA were within the generally accepted variation range for immunoassays [14]. A study by Livesey and colleagues [15] indicated that repeated thawing and freezing affected the insulin concentration in human plasma samples collected during intravenous glucose tolerance testing procedures. Insulin concentrations had already declined after the first freezing and thawing cycle and exhibited a continuous decline during following freezing and thawing cycles. A more recent study by Mechanic and colleagues [16] examined the effect of short-term refrigerated storage at ≤ −70 °C on insulin in human blood samples and found no significant effect in a time period of 72 h and also no significant effect of delays in freezing following centrifugation 2 h post-collection, whereas they observed declined insulin concentrations following delays in centrifugation. Furthermore, repeated freezing and thawing of serum did not affect the concentrations of insulin in serum samples from beagle dogs [17]. Borer-Weier and colleagues [13] evaluated the stability of equine insulin after freezing and thawing in their study and showed similar results to our study, with large ranges of differences in the samples analyzed with human-specific RIA^8^ and a high-range porcine insulin ELISA. One limitation in our study design, as well as in other previous studies, was that serum insulin concentrations were not measured directly after the sampling procedure, prior to first freezing, so the effect of the first, initial freeze remains unknown. Taking into account the variable effects of freezing and thawing on the insulin concentrations in the samples we analyzed, we recommend that samples are not frozen and thawed any more than necessary in order not to receive false results.

### Conversion factors for insulin measures

Various conversion factors have been established in the past due to the differences in insulin binding efficacy. Data had to be converted from μg/L into the commonly used SI unit μIU/mL to compare the results from ELISA with results derived from RIA and CLIA. In previous studies in which insulin analysis had been performed with the equine-optimized porcine-specific ELISA, data was either presented in μg/L [9, 10, 18] or converted into mU/L [19]. In the latter case, conversion was performed without an exact statement regarding the conversion factor, which was suggested to be 10 in an earlier publication by the same author [9]. In addition, the factor 28.69 (1 μg/L = 28.69 for μIU/mL) [20] for the conversion of human insulin was discussed. However, for the ELISA used in our study, the cross-reactivity with the human insulin molecule is only 22 %, as declared by the manufacturer. Therefore, the use of both factors for conversion of the data generated by the equine-optimized, porcine-specific ELISA might be questionable. Thus, another equine-specific conversion factor (1 μg/L = 117 mU/L) was given by the manufacturer for this assay [21] and was used for the calculation of data in our study.

### Comparison of methods and assay insulin antibodies

Equine insulin has a lower molecular weight (5.748 kDa) compared to human (5.808 kDa) and porcine insulin (5.778 kDa) and consists of 51 amino acids. The equine insulin differs from the human and porcine insulin molecule in the amino acid sequence [22, 23]. Even slight differences in the amino acid sequences between species and, thus, secondary structure can result in impaired antibody binding and can, therefore, cause variations in efficacy when measuring insulin by different immunoassay-based methods. Comparing the results of converted data, all three methods provided different insulin concentrations within the same samples. The CLIA results observed in this study were markedly lower than the results supplied by the ELISA and the RIA. Data analyses with Bland-Altman plots and Deming regression analyses indicated that there was no constant systemic error, meaning that one method measures consistently higher or lower than the other ones. The ELISA supplied higher insulin concentrations than the CLIA and the difference between both methods even increased with rising concentrations, indicating the presence of proportional error. The CLIA also provided lower results than the RIA for samples with concentrations under 100 μIU/mL, but when the concentration exceeded 100 μIU/mL, the CLIA supplied higher results for five samples compared to the RIA. These differences between insulin concentration results provided by the three assays may be caused by underlying antibodies used in the different assays. The ELISA and the RIA were based on antibodies directed against porcine insulin, which differs in amino acid 9 of the A chain (glycine in equine insulin and serine in porcine insulin), whereas human and equine insulin differ in amino acid 9 on the A chain and amino acid 30 of the B chain (alanine in equine insulin and threonine in human insulin) [22]. Differences at amino acid 9 on the A chain are not considered to be important for biological activity, but changes in amino acid 30 on the B chain were discussed to affect the 3D structure [23, 24]. Therefore, the markedly lower results provided by the CLIA may be caused by a loss of homology in this position and variation in 3D structure, which may impair sufficient antigen-antibody binding by steric interference. Furthermore, alterations in the antigenic epitope for the anti-human insulin antibody used in the CLIA may cause insufficient antigen-antibody binding. Variations in insulin concentrations, such as occurred in our study, have already been described by several authors for other assays and methods used frequently. Tinworth and colleagues [6] compared the equine-optimized, porcine-specific insulin ELISA and a modified version of the human-specific RIA^8^ in their investigations. Both assays gave different insulin concentration results for the same samples as well, and underestimated the insulin concentration compared to liquid chromatography and high-resolution/high-accuracy mass spectrometry (LC-MS) [6]. The LC-MS provides absolute amounts of insulin and could be assessed as a gold standard to measure insulin concentrations in blood samples. Due to a lack of LC-MS in our study, potential over- or underestimation of the assays examined could not be evaluated. Banse and colleagues [7] compared the human-specific RIA^8^ with another commercially available CLIA^9^ which is often used for the measurement of equine insulin in both clinical practice and research. Both the human-specific RIA^8^ and the CLIA^9^ resulted in poor recovery rates within each assay and showed poor accordance. They, furthermore, examined the recovery of equine insulin standard within both assays. The recovery rate was poor in both assays, but the CLIA^9^ investigated showed recovery rates of only up to 10 %. However, no equine insulin standard was available in our experiments; therefore, we could not examine the equine insulin standard recovery in the three immunoassays investigated in this study.

### Clinical relevance of valid insulin measures

The clinical relevance of result variation becomes obvious when focusing on basal samples. According to the current state in equine veterinary practice, analysis of basal samples from fasted patients as a screening method for IR and ID accounts for a large proportion of insulin measurements in veterinary diagnostics. In veterinary practice, basal insulin concentrations of 20 μIU/mL were stated as cut-off values to distinguish between healthy and insulin-resistant or insulin-dysregulated horses in the consensus statements of the American College of Veterinary Internal Medicine [12]. On closer inspection, this cut-off value was suggested as being valid for sample analyses resting upon the Coat-A-Count insulin radioimmunoassay^8^ (Siemens Medical Solutions Diagnostics, Los Angeles, CA), Immulite insulin solid-phase chemiluminescent assay^9^ (Siemens Medical Solutions Diagnostics) and DSL-1600 insulin radioimmunoassay (Diagnostic Systems Laboratory Inc, Webster, TX) by the authors of the consensus statement. However, this cut-off value is often used without consideration of the underlying analysis method. Thus, assay-dependent differences in the insulin concentration levels measured can cause false classification of patients. Calculations of indices, which are based on basal insulin and glucose concentrations for estimation of IR, were used more commonly [25] and, therefore, could result in false diagnosis of IR. Exact differentiation between 15 and 30 μIU/mL may be very important for the interpretation of basal samples. In six of the seventeen animals investigated, analysis of basal, fasted blood samples by the three assays investigated led to different classification as insulin-sensitive or insulin-dysregulated depending on the data used. One of our samples illustrates this problem. The CLIA supplied an insulin concentration of 7.8 μIU/mL, whereupon this horse is classified as insulin-sensitive when considering the cut-off value of 20 μIU/mL recommended by the American College of Veterinary Internal Medicine. By contrast, ELISA and RIA supplied insulin concentrations of 50.73 μIU/mL and 46.53 μIU/mL, respectively, after which this horse is classified as insulin-resistant or insulin-dysregulated on the basis of basal, fasted blood analysis. Both commercial laboratories we commissioned with the analyses of our samples provided laboratory-specific reference values for insulin in fasted serum blood samples of horses. The reference range stated for the RIA was < 20 μIU/mL in fasted samples and was < 23.4 μIU/mL for the CLIA. These findings indicate that even consideration of laboratory and assay-specific reference ranges leads to different classification of this horse, depending on the assay used to determine the basal, fasted blood insulin concentration. As a consequence, independent of laboratory and assay-specific reference ranges or cut-off values, a general harmonization of equine insulin analyses is urgently required to allow accurate and safe diagnosis of hyperinsulinemia as one of the leading symptoms in horses suffering from EMS.

## Conclusion

In conclusion, analyses of equine serum samples for insulin with three different immunoassay methods revealed three different results for the same sample. Adjustment of insulin measurements and methods is essential to allow the consistence of information of several studies and research approaches, enabling evidence-based criteria to compare insulin concentrations consistently between assays, studies and laboratories. Since harmonization is an interminable process, further assay comparison research studies would be helpful to allow the comparison of results and cut-off values determined with the use of one method to another. This study illustrates the importance of considering the insulin analysis method when results of different laboratories or studies are compared or interpreted for the diagnosis of insulin-related endocrine and metabolic pathologies.

## Abbreviations

CV: Coefficient of variation
EMS: Equine metabolic syndrome
Fig: Figure
ID: Insulin dysregulation
IR: Insulin resistance
ELISA: Enzyme-linked immunosorbent assay
RIA: Radioimmunoassay
CLIA: Chemiluminescent immunoassay
RUD: Recovery upon dilution
LC-MS: Liquid chromatography and high-resolution/high-accuracy mass spectrometry
SD: Standard deviation
Tab: Table

## Footnotes

1 Equine Insulin ELISA, Mercodia AB, Uppsala, Sweden.

2 Porcine Insulin RIA, Millipore, St. Charles, MO, USA.

3 ADVIA Centaur Insulin Assay, Siemens Healthcare Diagnostics GmbH, Eschborn, Germany.

4 Vacuette® Greiner Bio One, Frickenhausen, Germany.

5 Animal Insulin Control, 362 Mercodia AB, Uppsala, Sweden.

6 Diabetes Sample Buffer, Mercodia AB, Uppsala, Sweden.

7 GraphPad Prism, Version 6.02 for Windows, GraphPad Inc. La Jolla, CA, USA.

8 Coat-a-Count Insulin RIA, Siemens Medical Solutions Diagnostics, Los Angeles, CA, USA.

9 Immulite Insulin Assay, Siemens Healthcare, Malvern, PA, USA.
